# Supplementary material for: Autism Caregiver Coaching in Africa (ACACIA): Protocol for a type 1-hybrid effectiveness-implementation trial
Source: PLoS One. 2024 Jan 12;19(1):e0291883. doi: 10.1371/journal.pone.0291883 (PMC10786379; doi:10.1371/journal.pone.0291883)
Supplement: S1 Protocol — (DOCX) [file pone.0291883.s002.docx]

**Form FHS015: Research Protocol - Section C**

Title: Autism Caregiver Coaching in Africa

1. **BACKGROUND**

Globally there is growing recognition of the importance of strengthening the capacity of caregivers and providers to *implement community-based services that support improvements in quality of life of autistic people*.^1^ Early autism intervention is recognized as critical because when delivered by specialist interventionists it can significantly improve child and family outcomes, reduce the need for services at a later age, and be a cost-effective approach.^2, 3^ Most autistic people live in LMIC, where systems of care do not provide developmental screening or early intervention.^4, 5^ By 2050 forty percent of the world’s children will live in Africa.^8^ This demographic shift highlights the importance of *developing feasible early intervention services that can be scaled-up in existing systems of care*. Consistent with Global Brain FOA priorities and NIMH Strategic Objective 4, there is an urgent need to develop autism services that foster high quality equitable care.


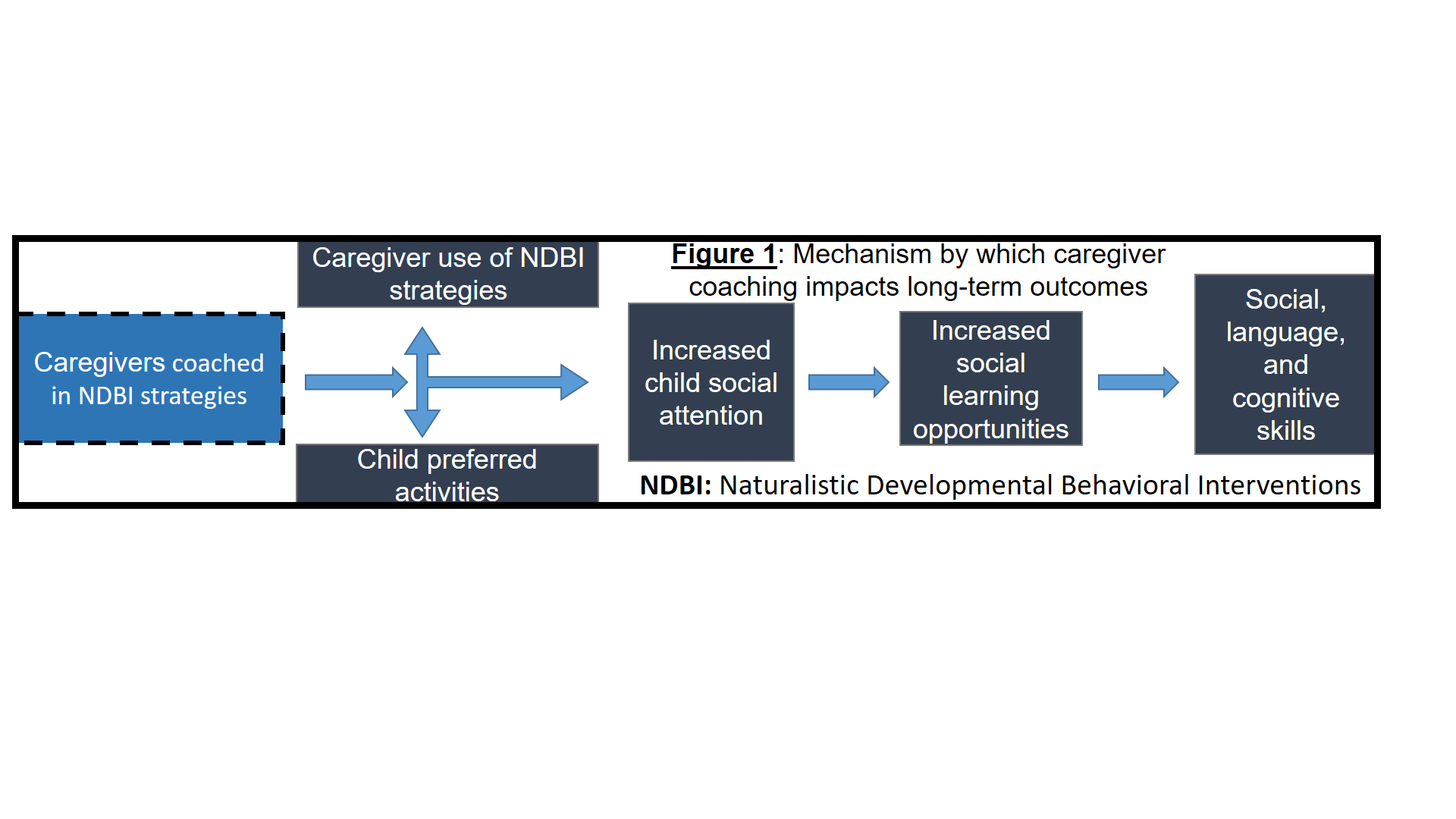
Research has demonstrated that caregivers can be coached to deliver early intervention strategies during everyday activities with their young autistic child.^10^ Low intensity caregiver coaching can impact caregiver-child interaction styles and improve child developmental outcomes.^23, 24^ **N**aturalistic **D**evelopmental **B**ehavioral **I**nterventions (**NDBI**) comprise a class of early intervention approaches that have been effectively delivered by caregivers.^11^ The Early Start Denver Model (ESDM) is an evidence-based NDBI.^12^ In the ESDM approach, strategies increase a child’s attention and engagement with their caregiver, thereby increasing opportunities for learning social and communication skills, leading to improvements in developmental outcomes (See Figure 1).

The efficacy of ESDM delivered via therapists, caregivers, and teachers has been evaluated in a wide range of cultures, including in Israel, France, Taiwan, Italy, China, and Japan, among others.^25-33^ Recent meta-analyses indicate that ESDM is efficacious for improving social, language, and cognitive skills.^34, 35^ A recent systematic review of caregiver-delivered social communication interventions for young autistic children concluded that ESDM qualified as an established evidence-based practice.^10^ While these studies indicate that caregiver-coaching based on ESDM has promise for addressing the needs of LMIC, the proposed research aims to address three important gaps in the existing literature. First, very few studies to date have included *diverse stakeholders*, including autistic self-advocates, in the research process.^36^ Such involvement is important for ensuring that the intervention is acceptable to the autism community.^37^ Second, caregiver coaches in most studies to date *have been highly trained specialists,* usually licensed in the fields of psychology and/or education*.* Lack of access to such specialists in most parts of the world requires that studies include task shifting to non-specialists as an implementation strategy. Third, in a meta-analysis of non-specialist delivered autism intervention, only two studies included participants from LMIC.^38-40^ Furthermore, in these two studies ‘non-specialists’ were certified teachers and therapists, a workforce unlikely to be scalable in Africa. In a study conducted in India, lay health workers under specialist supervision delivered 12 coaching sessions of a developmental intervention, demonstrating positive effects on children’s outcomes.^41^ This study provides preliminary evidence of the feasibility of non-specialist delivered early autism intervention in India, but implementation determinants will be different in other regions of the world, such as Africa. *Thus, our goal is to develop a feasible, scalable early autism intervention model in Africa by conducting research with culturally and linguistically diverse participants in community-based settings, that is inclusive of diverse stakeholder perspectives and incorporates task-shifting.*

In Africa, community health workers are often proposed as the non-specialists to target for task-shifting.^42^ In some LMIC, early childhood development services have been integrated into maternal and child community health worker programs.^43, 44^ *Challenges* with this approach include: limited community health worker training in early child development; difficulties managing competing demands resulting in poor quality of care and burn out; and lack of individualization of approach.^45-47^ This lack of individualization is particularly problematic for children with variable developmental profiles, which is seen in autism. *In the current proposal, we aim to address each of these challenges* by working with non-specialists trained in early child development, with dedicated time to coach, who work under supervision, and use an intervention that can be tailored to meet individual needs. Addressing these challenges will help create a non-specialist workforce that is well-positioned to deliver early autism intervention, thereby increasing the likelihood of long-term sustainability and scalability of the approach.

**Foundation for Proposed Project Built with NIMH R21 Funding**

*In South Africa few families with young children are able to access early autism intervention due to limited services and structural barriers*.^13, 14^ Those able to access services receive one 30-minute therapy session every 4–6 weeks. Caregivers are not included in sessions and typically asked to wait outside the room. Poverty and the legacy of apartheid remain significant structural barriers.^22^ Racial variation in expressive language has been documented in a neurodevelopmental clinic, with a significantly greater number of Black compared to White children non-verbal at diagnosis (94% vs 42%).^48^ The need for early autism intervention is increasing, with a documented 276% rise in the number of children on the waiting list for special education services.^49^

*Implementation context is critical to consider in early autism intervention*, particularly the ‘fit’ between intervention and context. Implementation frameworks specify the complex context for evidence-based practice implementation. The *Exploration, Preparation, Implementation, Sustainment (EPIS) framework* has been used to examine evidence-based practice implementation in publicly funded services.^21, 50^ EPIS highlights influential contextual factors within 4 phases: Exploration, Preparation, Implementation, and Sustainment and factors that hinder or support the implementation process when selecting, using, and sustaining evidence-based practice.

*A partnership between Duke University and the University of Cape Town (UCT) has laid the groundwork for an innovative and scalable coaching intervention.* The following achievements provide a strong foundation for our proposed project.


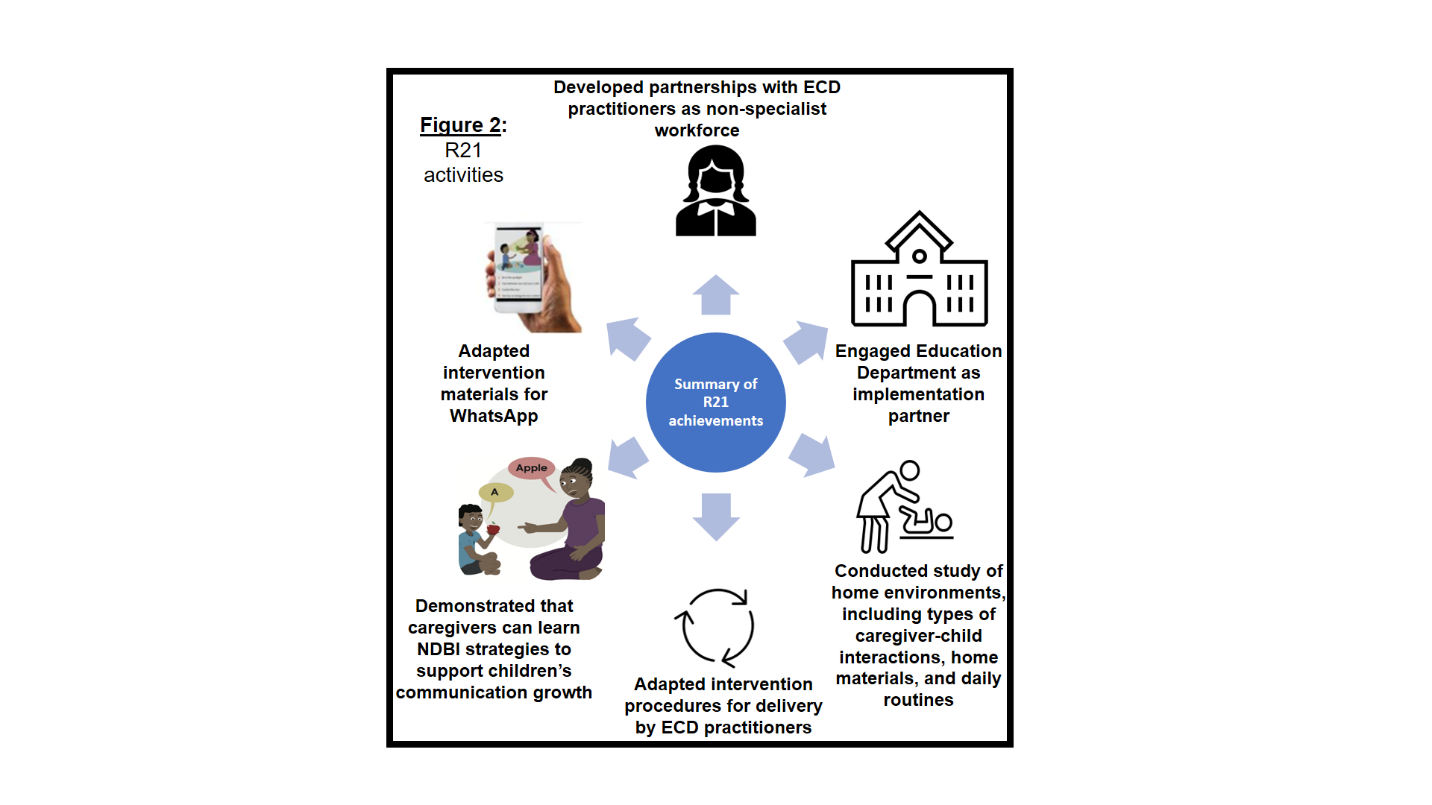
First, we identified Early Childhood Development (ECD) practitioners as the *non-specialist workforce* that could deliver the intervention.^14^ A partnership between this workforce and the National Education Department has been established.^19^ ECD practitioners are well-suited for this role as they are employed by the Education Department, trained in early child development, supervised, and supported by national policy.^51^ Alignment of the ECD workforce and Education Department at a national level is important for future scale-up. Second, we *identified the NDBI approach* based on local interest and available resources. Third, we evaluated the ‘*fit’ of NDBI-caregiver coaching with South African dyads*.^13, 15, 17^ We conducted interviews with caregivers and behavioral coding of caregiver-child interactions to understand interaction styles, and common daily routines in which intervention could be carried out. Fourth, we systematically *adapted ESDM procedures for effective delivery by non-specialists*. Fifth, we conducted a pilot study to *demonstrate that caregivers can learn NDBI strategies* when coached by ECD practitioners and that growth in child social communication occurs.^52^ Finally, mindful of the ‘digital divide’, intervention *materials were adapted to be shared through WhatsApp*, a low-cost messaging software.^18^ These achievements are shown in **Figure 2**.

1. **PURPOSE OF THE STUDY**

We now propose to build on this foundation by conducting a *type 1 hybrid effectiveness implementation trial of the intervention, delivered by non-specialists, within an existing system of care in South Africa*. The *main objectives* of this project are as follows:

**Aim 1**: Evaluate the effectiveness of an NDBI caregiver coaching intervention delivered by non-specialists relative to usual care for improving developmental outcomes for young autistic children in South Africa and assess the cost-effectiveness of this approach;

**Aim 2**: Identify the determinants (barriers and facilitators) that impact successful implementation of NDBI caregiver coaching delivered by non-specialists in South Africa; and

**Aim 3**: Build r**esearch capacity.** We aim to accelerate autism research capacity building in South Africa and extend it to Tanzania, through a tailored and systematic program of research training. Early career researchers at the University of Cape Town in South Africa, and Kilimanjaro Christian Medical Center in Tanzania will receive research training and be integrated into ongoing study activities. This aim will build sustainable autism research capacity and establish an African autism research network. We plan to build on our current relationships with families, practitioners, and policy makers by formalizing these relationships and including other key stakeholder groups such as South African autistic self-advocates through a *community-academic partnership, a key bridging factor in the EPIS implementation framework*.^21^

*This project also offers a unique opportunity to study variability in autism-related behaviors*. Exploratory components of Aim 1 will assess the degree to which response to intervention is moderated by dimensional child characteristics. In addition, using an innovative digital assessment method, changes in dimensional autism-related behaviors will be examined. Finally, cross-cultural differences in dimensional autism-related behaviors will be evaluated via comparison with existing quantitative phenotypic data gathered in U.S. studies conducted by the PI and co-investigators using the same measures.

1. **METHODOLOGY**
   1. **Study Design**

**Community-academic partnership.** In the proposed project, we will expand upon our work by conducting a *type 1 hybrid effectiveness implementation trial of caregiver coaching, delivered by non-specialists, within an existing system of care in South Africa*. With the hybrid type 1 design our primary objective is to determine the effectiveness of the coaching intervention, and our secondary objective is to identify implementation determinants for scale-up. To date, stakeholders have been integral to our work and engaged through public forums and research activities. We now plan to formalize these relationships through a community-academic partnership. *A community-academic partnership fits within EPIS as a bridging factor that can address potential ideological differences, help ensure research products are acceptable to end-users, and support longitudinal stakeholder engagement across the research cycle*.^21, 73^ The PI has extensive experience with forming such partnerships. At Duke, she currently directs the Duke Autism Center of Excellence Dissemination and Outreach Core, which includes a Community Engagement Advisory Board. This board is racially/ethnically diverse and includes autistic self-advocates, parents, educators, healthcare providers, and business leaders. This experience will be useful in designing and sustaining the community-academic partnership in the proposed project. Dr. Stahmer, an implementation scientist and expert in autism research is a consultant on the proposed project and will advise on the formation and measurement of partnership functioning.^74^ The Partnership will be comprised of the PI, Co-PI, the Education Department Director and the Deputy Chief of Inclusive and Specialized Education Support, and 12 stakeholders (caregivers, autistic adults, ECD practitioners, and school supervisors). We will ensure that the cultural and linguistic diversity of South Africa is represented within this partnership. Participation will include 4-5 meetings annually. Members will receive an honorarium for attending each meeting. Meetings will last 2-4 hours. Agenda items will vary by project stage and include: (1) Coaching materials and approach; (2) Assessments, including qualitative interviews; (3) Interpretation of findings; (4) Community dissemination messages and avenues; (5) Identifying what the research team should leave behind for the community; and (6) Emerging topics of interest. To ensure stakeholders, particularly autistic self-advocates and caregivers, feel they are authentic partners, we will offer pre-meetings to set the context, encourage participation, and provide strategies to facilitate discussion.

**Aim 1: Effectiveness trial evaluating the intervention model**

**Overview of intervention trial design and arm allocation.** *The goal of the intervention trial is to evaluate the effectiveness of NDBI caregiver coaching delivered by non-specialists relative to usual care for improving developmental outcomes for young autistic children in South Africa and assess the cost-effectiveness of this approach*.


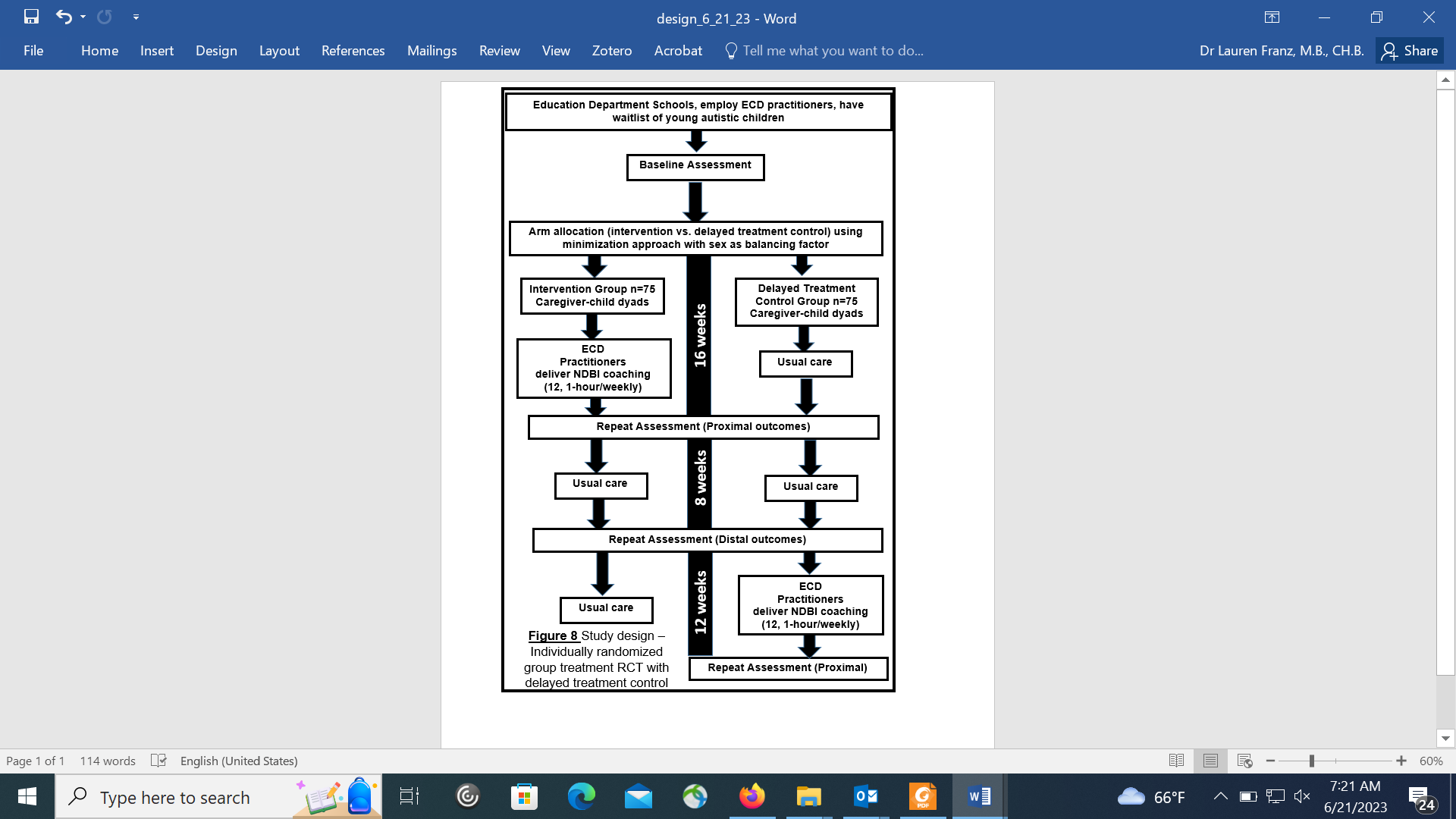
The study design will be an individually randomized group treatment RCT with a delayed treatment control group (**Figure 8**). 150 children, ages 18-72 months, with a DSM-5 diagnosis of autism spectrum disorder (ASD) and their caregivers will be recruited on a rolling basis, with arm allocation (coaching vs. delayed treatment control) conducted using a minimization approach with child sex balanced across groups.^75^ The intervention will be offered to the delayed treatment control group immediately after follow-up assessment (6-months after baseline). Offering the intervention to both groups is of ethical importance in an environment with few services and will enhance retention. Once control dyads have completed the intervention, the degree to which the trend in the control group is consistent with any treatment effect detected in the main trial comparison will be assessed.

Primary outcomes will be distal measures of communication abilities, as assessed by the VABS-3 - Communication Domain Standard Score and the Griffiths III - Language and Communication Developmental Quotient.^70, 71^ Key secondary outcomes will include proximal measures of the quality of the caregiver-child interaction which will be coded using the JERI coding scheme.^67, 68^ *Three dimensional measures that all assess level of autism symptom will be included*: the Social Responsiveness Scale-2 (SRS-2), the Childhood Autism Rating Scale, Second Edition (CARS2), and the S2K digital assessment tool.^58, 76, 77^

**Aim 2: Assess determinants that impact intervention implementation in South Africa**


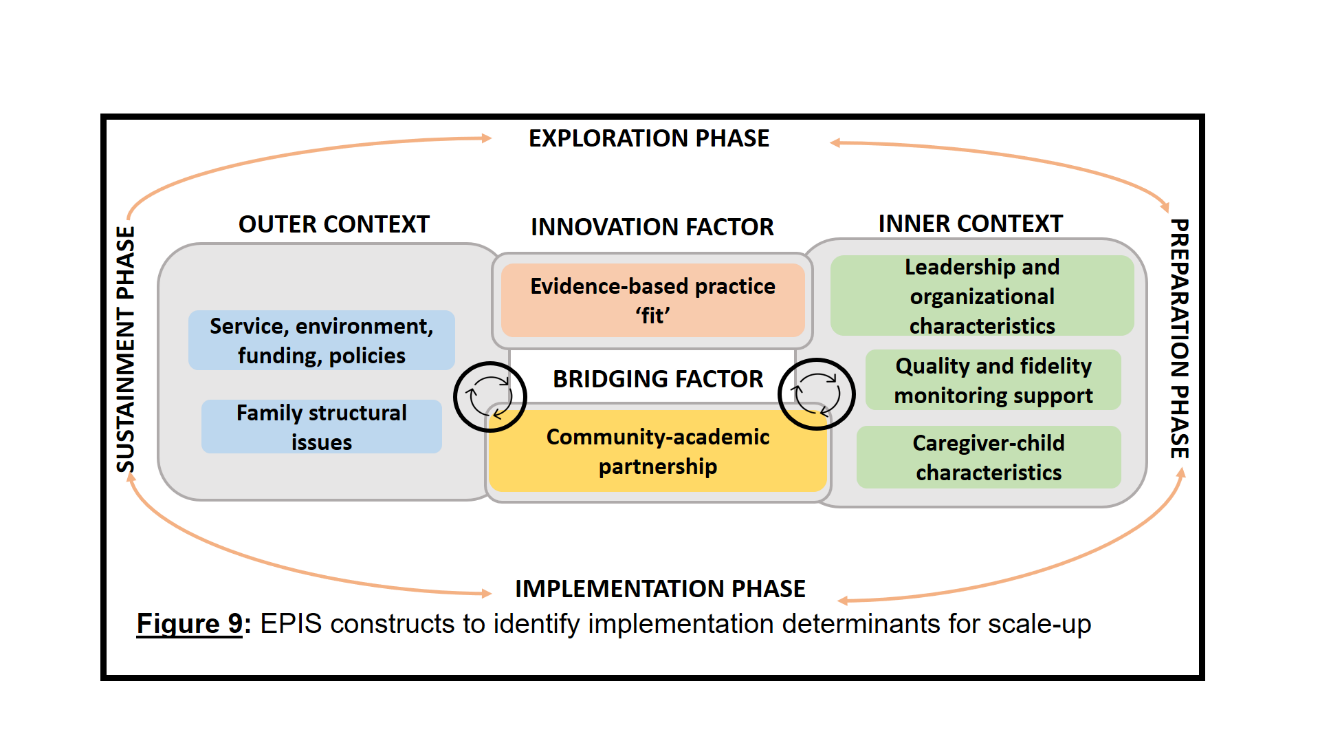
**Overview.** A key secondary objective of the hybrid type 1 design in the proposed study is to identify implementation determinants for scale-up. *Therefore, the goal of specific aim 2 is to identify the determinants (barriers and facilitators) that impact successful implementation of NDBI caregiver coaching delivered by non-specialists in South Africa*. With R21 funding, we identified implementation determinants and made several adjustments to mitigate barriers (e.g., created simple visuals and text of intervention session topics that could be sent to caregivers through WhatsApp).^16^

**Figure 9** outlines the specific EPIS framework outer and inner contextual, bridging and innovation factors, selected based on our formative work, that will be assessed in the proposed project**.** We will utilize a mixed methods approach to test whether preliminary determinants (e.g., intervention materials mismatched with the South African context) have been addressed and identify emerging implementation barriers and facilitators. Understanding implementation barriers even after adjustments in our approach, and ways to mitigate their impact, will *inform the development of implementation strategies that will be tested during future scale up.^89^*

**Aim 3: Build research capacity**

**Overview.** We aim to accelerate autism research capacity building in South Africa and extend it to Tanzania, through a *tailored and systematic program of research training*. Early career researchers at the University of Cape Town in South Africa, and Kilimanjaro Christian Medical Center (KCMC) in Tanzania will receive research training, as described below, and be integrated into ongoing study activities. This aim will build sustainable autism research capacity and establish an African autism research network.

**Autism research capacity in Africa.** In a published scoping review, the PI and Co-PI documented *significant geographic disparity in autism research output* in sub-Saharan Africa vs. other continents .^4^ We also documented significant geographic disparity in autism research output within sub-Saharan Africa. Of the 53 peer reviewed articles identified, the majority (n=28) were from South Africa. Notably, our review documented extensive autism knowledge gaps in early intervention, implementation science, high quality phenotyping and use of digital technologies. *Over the past 7 years, we have started to build autism research capacity in South Africa to begin to address these gaps*. The PI and Co-PI supported 6 training workshops on the topics of NDBI strategies, autism assessment and diagnosis, JERI behavioral coding, and dissemination and implementation science with over 100 participants attending from across South Africa. In addition, support for South African early career researchers at UCT has been provided in the form of structured mentorship for 5 Undergraduate, 1 Honors, 3 Masters and 1 PhD student. This support has resulted in poster and oral presentations at national and international conferences, a book chapter, and 5 manuscripts in peer-reviewed journals.^16-18, 65, 66, 72, 101-103^ The PI has conducted a site visit to KCMC in Tanzania where she identified Dr. Mmbaga as a research capacity building partner. Three KCMC early career researchers have already visited UCT on 2 separate occasions for introductory trainings in NDBI strategies and autism assessment and diagnosis.

**African autism research fellows** **training program.** We aim to establish an African autism research fellows training program with 3 main objectives: 1) *Expand research capacity-building at UCT*, 2) *Establish a research capacity-building program at KCMC*, and 3) *Establish a research capacity-building network in sub-Saharan Africa*. In Year 1, we will identify research fellows at UCT and KCMC. At UCT, candidates will be identified by Drs. de Vries and Franz through a competitive application process. *Five fellows will be selected at UCT*. Due to the diversity within South Africa, we will ensure that multicultural/multilingual trainees are selected. *Four candidates* will be selected at KCMC by Dr. Mmbaga. Because of the interdisciplinary nature of autism research, we will prioritize the selection of trainees from different professional backgrounds. We will also seek out the opportunity to identify and support early career autistic trainees. The 9 fellows will *each complete a scholarly research project* during their research fellowship. *Fellow activities and outcomes are specified in* ***Table 1***. Research products would vary based on site (UCT vs. KCMC) and level of expertise (Master’s vs. PhD level). In Year 1 we will conduct a *2-week intensive research skills training workshop* at UCT. Individual mentoring arrangements and project goals will be established during this 2-week training. Fellows will then be provided with ongoing, regular support and supervision (Zoom or in-person), guiding them through their research project. In Years 1-5, *3-monthly African autism* *research training fellowship seminars* (via Zoom) will be conducted for additional structured teaching and to review research progress. We will introduce *6-monthly* *African autism training network webinars* on autism research and research methods, including presentations by fellows. An additional advantage of these networking webinars would be to identify hubs for future autism research capacity building. *At UCT fellows will lead specific research activities* and enroll in Masters/PhD in Neuroscience degrees. Integrating degrees for UCT fellows is feasible because Dr. de Vries (Co-PI) directs the neuroscience training program. In Year 3 and 5 *KCMC Fellows will participate in a* *3-month intensive training* at UCT. Ongoing supervision will be provided for an additional 9-months on specific autism skills (e.g., autism assessment) following the intensive training, to support skill transfer. While at UCT, KCMC fellows will be integrated into ongoing activities to become familiar with the process of autism clinical trial implementation. An *annual 3-day networking meeting will be held at the Duke Center for Autism.* Meetings will focus on project activities and facilitate networking with Duke colleagues.


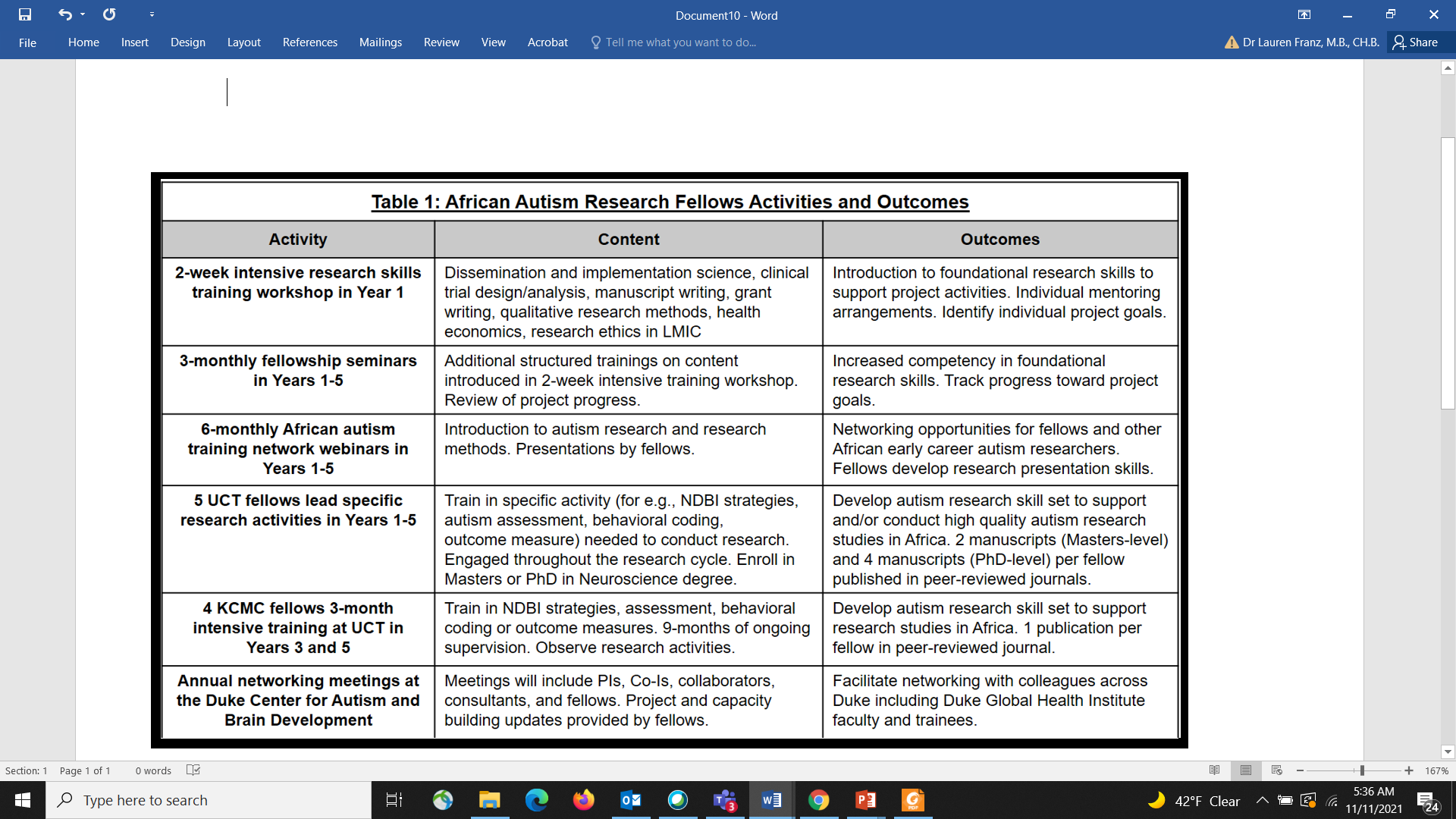


**Characteristics of the study population**

**Aim 1.** As we did previously with the R21, caregiver-child dyads will be recruited from school wait lists. Aim 1 inclusion criteria are: (1) child’s age is 18-72 months, (2) child meets DSM-5 criteria for ASD, informed by Autism Diagnostic Observation Schedule, Second Edition (ADOS-2) (*Note: DSM-5 is the primary diagnostic framework utilized in South Africa. The feasibility of the ADOS-2 in Afrikaans and Zulu, two high-frequency languages in South Africa, has been established*),^78, 79^ (3) child’s caregiver speaks isiXhosa, isiZulu, Afrikaans, or English (97% of Western Cape languages), (4) child’s race is African or Coloured (South African term for mixed race), (5) lives in recruitment area, and (6) caregiver is ≥18 years. Exclusion criteria are: (1) genetic disorder of known etiology (e.g., fragile X syndrome), (2) significant sensory or motor impairment that would preclude use of the play materials, (3) major physical abnormalities that would interfere with participation in the intervention, a (4) history of serious head injury and/or neurological disease, and (5) caregiver indicates they will be unable to attend assessments and 12 sessions. We will enrol 150 caregiver-child dyads in Aim 1 (300 participants).

**Aim 2.** Participants will include: (1) *those involved in intervention implementation through participation in the intervention trial; and (2) community-academic partnership members*. Specifically, caregivers who receive NDBI coaching, ECD practitioners who conduct coaching, the Education Department autism outreach team who supervise ECD practitioners, and Education Department leadership will be included. These participants will be directly involved in intervention implementation and be able to provide input on the specific outer and inner contextual, and innovation factors outlined in the EPIS framework**.** Community-academic partnership members include the PI, Co-PI, Education Department leadership, and other stakeholders (for e.g., caregivers and autistic adults). These participants will provide input on functioning of the community-academic partnership, a key bridging factor in EPIS. Education Department leadership, specifically the Director and Deputy Chief of Inclusive and Specialized Education Support will be in both participant groups. We will enrol 20 participants for Aim 2.

- 1. **Recruitment and Enrolment**

**Recruitment plan.** The proposed project will utilize the same recruitment procedures (for Aim 1 participants) that were utilized in the Global Brain R21, with caregiver-child dyads being recruited from school wait lists. Eligible children with autism within the study age range of 18-72 months will be identified by partner schools (Western Cape Education Department Schools: Mary Harding, Lentegeur, Beacon, and Glenbridge). Partner sites have records of children with autism, who have received a clinical diagnosis, and are waiting for public sector special education services. Across the four recruitment sites, 217 autistic children would likely be eligible to be recruited for initial screening, based on autism diagnosis and age. We plan to recruit 120 caregiver-child dyads in total. Based on experience from ongoing R21 activities we anticipate that at minimum 70% of these eligible subjects will meet full inclusion/exclusion criteria. This will most likely be related to caregiver availability to attend all intervention/assessment sessions. An exclusion criterion is a caregiver-child dyad that is unable to attend baseline and follow up assessments, as well as 12, 1-hour, caregiver coaching sessions. In order to account for the estimated 20% attrition, and ensure a final total sample size of 120 caregiver-child dyads, 150 caregiver-child dyads will initially be recruited. Enrolment will occur over a 39-month period beginning in month 5. Families will be invited to participate in the study by school staff using IRB approved recruitment materials. Those who are interested will be contacted by a research team member who will explain the study and gather the screener questionnaire data. If the child and caregiver meet checklist criteria and caregiver provides telephonic informed consent, the child and family will be enrolled. Data from the child, the caregiver-child dyad, and the caregiver are relevant to the study questions. Therefore, the parent/legal guardian will sign consent both on behalf of their young child with autism, and themselves. The caregivers and their children are recruited as a ‘unit’. Following screening consent, the Project Coordinator, in addition to screening the autistic child with for most inclusion and exclusion criteria, will conduct an additional screening of the caregiver. As part of the baseline assessment an ADOS-2 will be administered to inform DSM 5 autism spectrum disorder diagnosis. Participants will be informed throughout the recruitment process and study sessions that participation is voluntary and that they may refuse to answer any question for any reason. In addition, the caregivers of their child may refuse to participate or withdraw from the study at any time without penalty or loss of benefits to which they are otherwise entitled. In cases where a dyad requests change in caregiver due to change in personal circumstances negatively impacting their ability to attend sessions or assessments we will work with the family to identify and consent a caregiver that meets inclusion criteria.

Aim 2 participants will be purposively sampled and include (1) those involved in intervention implementation through participation in the intervention trial; and (2) community-academic partnership members. Specifically, multilevel stakeholders who receive (caregivers in the intervention arm), deliver (ECD practitioners), supervise (Education Department autism outreach team) NDBI coaching across 4 school partner sites. Furthermore, because the Education Department is our implementation partner, Education Department leadership will be included as participants. Community-academic partnership members include the PI, Co-PI, Education Department leadership, and other stakeholders (for e.g., caregivers and autistic adults).

**Retention strategies.** In the intervention study, based on our experience in R21, we estimate 20% attrition. Thus, 150 caregiver-child dyads will be recruited. Using strategies established during the R21 we will create a tracking log to document the dates of study visits, including intervention sessions and assessments, as well as other notes related to participant attendance and retention. A centralized tracking log keeps all study staff at UCT and Duke up-to-date and allows for the Project Coordinator and interventionists to track when visits are scheduled and make reminder calls in a timely manner. The tracking log also allows for study staff to document notes about participant circumstances that may influence scheduling (e.g., vacation dates, work schedules), note reasons for missed study visits, and record staff attempts to contact participants. These details all help inform individualized strategies to retain participants.

- 1. **Research Procedures and Data Collection Methods**

**Aim 1.** The majority of measures in the proposed project were included in the R21 and are used clinically in South Africa. *The primary objective is to evaluate the effectiveness of NDBI caregiver coaching on distal developmental outcomes relative to usual care*. Co-primary distal outcomes will be change from baseline to 6-months (2-months post-intervention) on the VABS-3 Communication Domain Standard Score and the Griffiths III Language and Communication Developmental Quotient. VABS-3 is a semi-structured, clinician-administered, caregiver interview that assesses child adaptive behaviors.^70, 80^ Griffiths III is a clinician-administered child developmental assessment.^71^ Both the VABS-3 and Griffiths III will be administered by trained clinicians blinded to treatment condition. *The secondary objective is to evaluate the effectiveness of NDBI caregiver coaching on proximal outcomes relative to usual care***.** The key proximal outcome will be change from baseline to 4-months (immediately post-intervention) on caregiver strategies measured by the sum of 5 caregiver items from the JERI, a behavioral coding measure (Scaffolding, Following the child’s interests, Affect, Language facilitation, and Use of communicative temptations).^67, 68^ These items have been selected because they are aligned with common fidelity elements of an NDBI approach.^81^ A second proximal outcome will be caregiver change on the ESDM fidelity scale, which assesses the degree of fidelity with which the caregiver is using intervention strategies.^69^ Both of these proximal measures will be assessed during a 6-minute free play session during which a standardized set of materials will be provided and caregivers are asked to interact with their child as they naturally would. These sessions will be videotaped and coded by blinded research-reliable raters. *The tertiary objective is to evaluate the proposed mechanism by which NDBI caregiver coaching impacts distal outcomes*. Specifically, it is proposed that NDBI caregiver coaching will lead to increased use of caregiver NDBI strategies (defined in secondary objective above), which in turn will support child language acquisition (defined in the primary objective above). *Three exploratory objectives are included*. *First*, we will evaluate the degree to which response to intervention is moderated by dimensional autism-related behaviors and caregiver characteristics. This will be assessed both quantitatively and qualitatively. Quantitative child measures include: the SRS-2, a caregiver-rated dimensional measure of child social ability;^76^ and the CARS2, a clinician-rated dimensional measure of severity of autism-related behaviors,^77^ Caregiver measures include the Parenting Stress Index, which assesses caregiver stress; and the Caregiver Sense of Competence scale, which assesses caregiver competence.^82, 83^ Qualitative individual interviews with caregivers will assess variability in interpretation of the intervention, and how such variability might relate to variability in child characteristics, and intervention response. *Second*, using the S2K App, we will assess changes in dimensional quantitative measures of social attention, affective expression, attention to speech, fine motor skills, and postural control.^58^ *Third*, we will conduct cross-cultural comparisons of dimensional autism-related behaviors using the S2K App, via comparison of South Africa data with existing phenotypic data gathered in U.S. The S2K app has been delivered to 769 children in the same age range as part of an NIH-funded Autism Center of Excellence research program at Duke. For the cost-effectiveness analysis three different types of costs will be measured for each participant at 4-and 6-month marks, including: (1) direct intervention costs (e.g., coaches’ time and supervision), (2) direct non-intervention costs (e.g., travel), and (3) indirect costs (e.g., wages forgone to take child to coaching).

**Aim 2.** Qualitative interview guides from the R21, tailored to each stakeholder group, will be adapted to explore identified EPIS constructs. Qualitative interviews (individual interviews or focus group discussions) will be conducted by study staff fluent in local languages who will be supervised by study PI/Co-PI. Each interview will be audio-recorded, transcribed verbatim and cross-checked. In addition, quantitative measures, described below, will assess specific EPIS constructs and be administered to participants on a tablet with staff support as needed.

**EPIS constructs and measures.** *Service, environment, policies, and funding*: External educational supports, and learning collaborative environments could sustain the intervention and support scale-up.^21^ In the proposed project we will build capacity of the Western Cape Education Department multi-disciplinary autism outreach team to supervise ECD practitioners by training this team to certification in ESDM. In Year 3 of the proposed project, qualitative individual interviews (with Education Department Provincial leadership) and focus group discussions (with Education Department autism outreach team members) will explore the feasibility of external training, ongoing support and monitoring, and funding avenues that could facilitate these efforts. Education Department policies, reports and regulations will be reviewed to identify structural funding and ongoing support opportunities.

*Family structural issues:* Health disparities result from complex interactions between socioeconomic and system-level factors.^90^ Structural challenges may impact families’ ability to learn coaching strategies. Qualitative individual interviews with caregivers who receive coaching will explore: social support, culture and language, stigma, caregiving practices, and early intervention preferences.^13, 16^ Quantitative measures will assess demographics including socioeconomic status, and caregiver strain, which will be assessed using the Caregiver Strain Questionnaire, a 21-item measure of self-reported strain experienced by caregivers and families of youth with emotional challenges.^91^ Data will be collected during proximal repeat assessments (See **Figure 8**).

*Evidence-based practice ‘fit’:* Stakeholder perceptions of an intervention’s contextual ‘fit’ can impact implementation and scale-up.^50, 92^ Qualitative individual interviews with caregivers; and focus group discussions with ECD practitioners and the outreach team will assess perceived ‘fit’ of the intervention with stakeholder needs, factors that facilitate or hinder implementation, and suggested improvements. Caregiver data will be collected during proximal repeat assessment (See **Figure 8**). Data will be collected from other participants during Year 3 of the proposed project.

*Community-academic partnership:* This partnership is a key bridging factor in EPIS.^21, 73^ We will evaluate specific interpersonal and operational factors that facilitate or hinder initiation and sustainment of this partnership. In Years 2 and 4 members will complete a quantitative survey that includes a list of facilitating and hindering factors. This survey was developed by Gomez and colleagues, and items were informed by a community-academic partnership systematic review.^74, 93^ Members will select whether each facilitating factor was ‘present’ or ‘not present’ during the preceding year of the community-academic partnership. For each factor selected as ‘present’, participants will rank how influential they believe the factor was in facilitating collaboration. This process will be repeated for the list of hindering factors. A qualitative individual interview will then be completed by each member to expand upon specific facilitating and hindering factors selected on the survey.

*Leadership and organizational characteristics*: School leadership can support staff buy-in and provide logistical supports that impact implementation.^16^ In Year 3, focus groups with ECD practitioners and the outreach team will assess school organizational leadership, absorptive capacity and coaching receptivity. Organizational characteristics will also be assessed quantitatively with the Implementation leadership scale, a 12-item measure assessing leader’s support for evidence-based practice implementation; and the Implementation climate scale, a 18-item measure assessing the extent to which an organization prioritizes and values successful implementation of evidence-based practices.^94, 95^

*Quality and fidelity monitoring support*: Provider’s ability to implement a program as intended is impacted by ongoing support.^16, 96^ In Year 3 of the proposed project, focus groups with ECD practitioners and the outreach team will assess internal school supports that may facilitate fidelity monitoring and stakeholder experiences of the fidelity monitoring system. Coaching fidelity will be assessed quantitatively, using the ESDM coaching fidelity scale, from video-recorded coaching sessions, coded by blinded research reliable raters.^97^ If challenges with key coaching behaviors are identified, ongoing supervision will target these specific coaching behaviors (for e.g., being ‘collaborative’, or ‘reflective’).

*Individual characteristics*: Attitudes towards coaching may sustain practice and influence adoption decisions.^16, 98, 99^ Qualitative individual interviews with caregivers will assess attitudes towards coaching. Caregiver data will be collected during proximal repeat assessments (See **Figure 8**). In Year 3, focus groups with ECD practitioners and the outreach team will assess attitudes towards coaching, and perceived need to change practice.

- 1. **Data Analysis**

**Data Analysis – Aim 1.** For the *primary objective*, co-primary outcomes include the VABS-3 Communication Domain Standard Score and the Griffiths III Language and Communication Developmental Quotient. For each outcome, an Analysis of Covariance (ANCOVA) type model will test for the effect of NDBI coaching at month 6 compared with usual care, adjusted for baseline differences between child intellectual ability as assessed by the Griffiths III Foundations of Learning Subscale with random effects to account for the partially nested design and for correlation within children over time. The Foundations of Learning Subscale assesses attention, memory, and processing speed. In addition, we will track all non-study intervention services and adjust the model for number of service hours received. The primary analysis will simultaneously model data from baseline, post-intervention, and follow-up assessments, before the delayed treatment control receives intervention. Once control dyads have completed the intervention, we will conduct additional analyses to assess the degree to which the trend in the control group is consistent with any treatment effect in the main trial comparison. For the *secondary objective*, each outcome will be analyzed separately using an ANCOVA model using the same form as for each of the primary outcomes. For the *tertiary objective* a mediation analysis will be performed using 4-month uptake in caregiver strategies as a mediator on the pathway between the intervention and 6-month co-primary distal outcomes.^84^ For the *1^st^ exploratory component*, each potential moderator will be considered separately by adding the following fixed effect terms to each ANCOVA model for each of the two primary outcomes: the potential moderator and its interaction with the NDBI treatment indicator. Qualitative interviews will be analyzed using an iterative coding and review process informed by grounded theory.^85^ For the *2^nd^ exploratory component*, changes in S2K dimensional quantitative measures of autism-related behaviors will be evaluated longitudinally (change over time) through data visualization (e.g. scatterplots) and group comparisons using a two-tailed Mann-Whitney U test. For the *3^rd^ exploratory component*, that includes cross-cultural comparison, visualizations and the Mann-Whitney U test will be used to compare the distribution of autism-related behaviors between the US and South African samples. For our primary analysis, our power calculations follow the approach of Moerbeek & Teernestra for an individually randomized group treatment design with clustering in one treatment arm^71^. Assuming 20% attrition in both arms, for 80% power and an alpha of 0.025, we need to enroll 75 dyads in the intervention and 75 in the control arm, for a total sample size of 150. For additional details see Human Subjects Section: Statistical Design and Power.

**Cost effectiveness.** To evaluate cost effectiveness we will adopt a systems and modified societal perspective, and limit the time horizon to the period of study enrolment.^86, 87^ Effectiveness will be measured as 4-month and 6-month change from baseline in VABS-3 Communication Domain Standard Score and the Griffiths III Language and Communication Developmental Quotient. Three different types of costs will be measured for each participant at 3-and 6-month marks, including: (1) direct intervention costs (e.g., coaches’ time and supervision costs), (2) direct non-intervention costs (e.g., travel), and (3) indirect costs (e.g., wages forgone to take child to coaching). Cost data will be collected in local currency units and translated to 2020 US dollars for analysis, while a discount rate of 3% will be applied to all cost and outcome measures. Cost-effectiveness will be assessed as Incremental Cost-Effectiveness Ratios, meaning difference in costs between intervention and standard care divided by difference in effectiveness.^88^ We will conduct intent-to-treat analysis and limit the time horizon to intra-trial costs and outcomes at 4-and-6-months. We will estimate incremental costs per unit increase in co-primary outcomes. For each cost and outcome measure, we will estimate means and standard errors separately for both groups. Incremental costs will be estimated as the difference in costs between coaching and usual care, while incremental effectiveness will be measured as the difference in each co-primary outcome between coaching and usual care. To test the robustness of the estimates, we will conduct one-way and probabilistic sensitivity analysis, and plot acceptability curves to highlight levels of confidence in the Incremental Cost-Effectiveness Ratios.^86^

**Data analysis – Aim 2.** We will utilize a mixed methods approach to test whether preliminary determinants have been addressed and identify emerging implementation barriers and facilitators. *Qualitative analysis will use an iterative coding and review process informed by grounded theory*.^85^ Qualitative data will be coded independently by two team members at a general level to condense data into analyzable units. Responses will be assigned codes based on a priori or emergent themes. Disagreements will be resolved by transcript review and discussion. A final list of codes will be developed through consensus. Axial coding will then be used to generate categories arranged in a treelike structure connecting segments into separate nodes, describing the relationship between codes and subcategories. Nodes and trees will be used to create a taxonomy of themes. Qualitative coding will then be quantified using frequency counts to identify primary themes. *Quantitative measures will be summarized using descriptive statistics*. T*hree main strategies will be used to integrate quantitative and qualitative data*.^100^ (1) Merging qualitative and quantitative data will occur through triangulation in which results are placed side by side to determine whether each provides the same answer to the same question (*convergence*; e.g., to what extent do provider qualitative data concur with quantitative data regarding school leadership). (2) Results will be linked when the former is used to provide explanations for unanticipated findings produced by the latter (*expansion*; e.g., survey data will be used to assess the prevalence of emergent barriers to implementing coaching observed in focus groups). (3) Results of qualitative analysis can be embedded within the analysis of quantitative data to contextualize quantitative results (*complementarity*).

- 1. **Data Safety and Monitoring Plan**

The proposed research will establish the following procedures, in compliance with NIH requirements, to ensure participant safety and protect the validity and integrity of data.

# Roles and Responsibilities

Dr. Franz (PI) and Dr. de Vries (Co-PI) will be primarily responsible for oversight of the conduct of the study, and will monitor the safety and data integrity for the project. Dr. Franz and Dr. de Vries will meet regularly with the study team to discuss study progress, including any adverse events or data integrity concerns. All deviations and/or corrective actions requiring reporting will be submitted to the University of Cape Town Human Research Ethics Committee (HREC) and the Duke Health System Institutional Review Board (DUHS IRB).

In accordance with institutional policy, a Clinical Quality Management Plan (CQMP) will be developed prior to institutional approval of the study protocol to independently oversee study conduct. The policy directs that an independent quality management (QM) reviewer, designated by the Duke Psychiatry Clinical Research Unit, will monitor regulatory and consented subject records at least twice a year. The CQMP reviews are filed with the Duke Office of Scientific Integrity for oversight, to insure that adequate corrective action plans have been implemented, appropriate notifications have been submitted to regulatory authorities, and that non-compliance does not become recurrent. A summary report of conduct concerns within each Clinical Research Unit is reported to institutional leadership on a quarterly basis.

At least twice a year, and any time an adverse event occurs, Dr. Franz and Dr. de Vries will conduct a data and safety review. During this review process, the PIs will evaluate any adverse events and determine whether the event changes the risk/benefit ratio of the study and whether modifications to the protocol or consent process are required. Throughout the project, they will maintain a “critical incident journal” that will contain information such as the nature of the incident, the personnel involved, solutions considered, and the final action implemented to resolve it. When necessary, they will seek recommendation from the University of Cape Town Human Research Ethics Committee (HREC) and the Duke University IRB and the Duke Office of Audit, Risk and Compliance. In addition, the accuracy and integrity of the data collected will be evaluated.

The University of Cape Town Human Research Ethics Committee (HREC) and the Duke IRB will also review this protocol on a yearly basis (or more frequently if deemed necessary) as part of the continuing review process. At this time, they will complete the following: (1) reassess the risks and benefits to participants, the informed consent process, and safeguards for human subjects; (2) review participant enrollment and retention; (3) consider any new scientific or therapeutic developments that might impact the safety of participants in the study; and (4) review any adverse events. Changes to the DSMP will be made as needed.

# Trial safety

Adequate measures will be used to insure that subjects enrolled in the study will have the opportunity to evaluate and review the consent for the study, and that privacy will be maintained during the conduct of interventional sessions and study related surveys and interviews. The primary risks for this investigation are a loss of confidentiality; all providers, including Early Childhood Development Practitioners, will adhere to standard practices and requirements for privacy and the reporting of significant safety concerns such as child abuse or suicidal ideation.

# Reporting procedures for adverse events

The investigator will adhere to the University of Cape Town Human Research Ethics Committee (HREC) and Duke IRB policies on unanticipated problems involving risks to participants or others. The following problems would require prompt reporting to the IRB:

- Any serious or non-serious adverse event that is unanticipated and indicates that the research places participants or others at greater risk of harm than was previously known or recognized.
- Information that indicates an adverse change to the risks or potential benefits of the research.
- Allegation of non-compliance with protocol requirements.
- Breach of privacy or confidentiality.
- Incarceration of a participant in a protocol not approved to enroll prisoners.
- Sponsor imposed suspension for risk.
- Complaint of a participant if it indicates unexpected risks or cannot be resolved by the research team.
- Any protocol deviation or violation that harmed participants or others, that indicates participants or others may be at increased risk of harm, or that compromises the integrity of the research data.
- Any protocol changes made without prior IRB approval to eliminate apparent immediate harm.
- Any safety reporting requirements specified by the IRB as a condition of approval.
- Any other problem the investigator considers to be unanticipated and indicates that participants or others are at increased risk of harm.

Reporting to institutional ethical review boards will occur at the following times: Within 24 hours upon learning of an unanticipated study-related death, study staff will notify the University of Cape Town Human Research Ethics Committee (HREC) and the Duke IRB via email or fax and provide a brief summary of the event. Within 1 week, study staff will submit a Safety Event submission to the University of Cape Town Human Research Ethics Committee (HREC) and the Duke IRB. Study staff will notify the University of Cape Town Human Research Ethics Committee (HREC) and the Duke IRB within 5 business days of an investigator becoming aware of a serious adverse event by a Safety Event submission in the iRIS IRB application.

Data Management and Quality Assurance

Electronic data, including audio recordings and transcriptions, will be stored on a secure server managed by the Duke Health System (DHTS), protected by two factor authentication. Integrity and accuracy of recordings and data will be monitored on an ongoing basis at the time of transcription. Quality assurance data for intervention sessions, which will be video-recorded, will include fidelity coding of all sessions by certified Early Start Denver Model therapists.

# Data management

The Duke PI, UCT Co-PI, and Study Coordinator will oversee the data management protocol and established Standard Operating Procedures (SOPs) for data collection, quality control, and data extraction and transfer.

Our research team is very aware of the importance of maintaining strict confidentiality and has extensive experience dealing with sensitive information. The following precautions will protect the privacy of participants and maintain confidentiality of research data during collection and transfer. All study staff will be well trained and will receive ongoing supervision in confidentiality and data security procedures, specifically in ethical conduct, confidentiality protection, mandated reporting, and other topics of human participant protection.

All data will be coded with a unique participant ID number. The key linking participant names and ID numbers will be stored in a separate password protected document on a password protected computer, to which only essential study staff will have access. Non-electronic data will be securely stored (with unique study ID only) in locked file cabinets in locked offices in the Division of Child and Adolescent Psychiatry at the University of Cape Town. Access to data storage areas will be restricted to essential study staff. Data will be securely stored. For electronic data storage management: Duke Health Internal will manage the infrastructure where electronic research data is stored (Duke REDCap). Data will also be stored within a folder on a Duke Health Server (Server and Folder Name\\duhsnas-pri.dhe.duke.edu\dusom_cabd\All_Staff\Franz South Africa). The data will be stored there, and relevant study staff will access the drive through a secure VPN connection. No electronic data will be stored on a laptop or external hard drive. Data will be collected/processed on an encrypted smart phone/tablet (Sense2Know App). Mobile devises are developed for this study as part of the study specific aims, and the software required for this study is only available on mobile platforms. Intervention sessions and some assessments are video recorded for later behavioral coding purposes. Intervention session video data will be transferred to a secure, HIPAA-compliant project folder on box.com at Duke University through Box Sync, and deleted from the recording equipment post-transfer. Individual interviews/focus group discussions are audio-recorded for later qualitative coding purposes. NVivo coding software will be used to analyze qualitative data. Access to data storage areas and computers will be restricted. All tablets and laptop computers will be running software that will allow the team to remotely erase any devices in case of theft.

1. **Description of Risks and Benefits**

Minimal risks are foreseen in the study. Participation will be completely voluntary and no associated risks are anticipated with participation or withdrawal from the study.

**Potential Risks.** The study population includes young children autism and their caregivers. Potential risks to participants in this study are the following: 1) negative consequences if confidentiality of information obtained in the study (including subject identity as a research participant or information collected during assessments) were compromised; and 2) distress or emotional reactions related to the qualitative topics, study assessments and/or intervention topics or activities. To protect against distress or emotional reactions related to assessments or intervention sessions, the clinicians who complete the assessments and supervise the intervention sessions, will: (1) be carefully trained; and (2) have extensive experience in working with young children with developmental challenges. Our goal is for the experience to be a positive one from which the caregiver and child will derive immediate benefits (e.g. opportunity to talk about their child, obtain a clinical report, engage in play activities, and caregiver would gain intervention skills).

**Additional Protections for Vulnerable Subjects.** Given that all child participants are under the age of 18, a signed parental permission form will be obtained from the parent or legal guardian of the child. The informed permission forms will be reviewed with the parent/guardian. The researcher obtaining consent will describe the study, procedures, risks and benefits to parents with a special focus on making procedures as anxiety-free as possible. Per University of Cape Town policy (study site), assent of minors is only required for children ages 7 and older, therefore, assent will not be obtained from participants in this study as all children are younger than age 6. The researcher obtaining Informed Consent will thoroughly review the consent form with the participant’s caregiver, including study procedures and potential risks and benefits of study participation. Informed consent processes will also be carefully documented. The caregiver will be encouraged to ask questions throughout the process. It will be emphasized that research is voluntary and that the participant can opt out of this project at any time without jeopardizing his/her treatment. The researcher will then answer any questions regarding the study and remind the participant and parents that participation in the study is voluntary and that it can be terminated by the participant at any time with no obligation to continue and no penalties whatsoever. All staff are highly experienced in working with children with developmental disabilities and use a variety of procedures to increase the comfort and safety of all children throughout the study.

**Potential Benefits of the Research.** The risk/benefit ratio for this study is relatively low, because (1) all data collection procedures are non-invasive, (2) no medications or other medical treatments will be provided as part of the intervention, (3) all study procedures will be voluntary, and (4) no deception or coercion is involved. All procedures will be performed by trained study staff to minimize risks, discomfort, and adverse events. Some study participants may benefit directly from participating in the study. Participants who require administration of diagnostic and/or developmental assessment will receive a written report and oral feedback on these assessments. Caregivers who are seeking clinical services for their child will be provided with referral information for appropriate community services. As an intervention study, there is the potential for direct benefits to participants who will receive the intervention. We anticipate that children with autism who receive the intervention will demonstrate gains joint attention, affective engagement, and receptive and expressive language. In addition, we anticipate that their caregivers will experience a reduction in parenting stress and a greater sense of competence as it relates to parenting their young autistic child.

1. **Informed consent process**

Before the study is initiated, it will be approved by the University of Cape Town Human Research Ethics Council (HREC)/IRB and the Duke Health’s Institutional Review Board (IRB). Each subject’s parent or guardian will provide consent for child participation in this study.

Participants who meet study inclusion criteria and are interested in learning more about the study will be informed of the study in detail in their preferred language, using language accessible to the study population. Study staff are able to speak isiXhosa, isiZulu, Afrikaans, and English. Patients who are eligible and agree to enroll in the study will read and be read aloud the consent form in their preferred language. Aim 1 participants (caregiver-child dyads) will be explicitly informed that the study in which they are about to enroll is an intervention study which will require participation for baseline and follow up assessments, as well as 12, 1-hour, caregiver coaching sessions. They will also be made aware that this study will involve questions about and assessments of their child’s development, interactions they have with their child, and that they will receive coaching in strategies to engage their young child with autism. All participants will be informed that the caregiver-child interaction at baseline, follow up and all intervention sessions will be video recorded, and in-depth interviews conducted at follow-up will be audio recorded. Furthermore, the study design will be an individually randomized group treatment RCT with a delayed treatment control arm. The intervention will be offered to the delayed treatment control group immediately after the follow-up assessment is complete (6 months’ post-baseline). Offering the intervention to both groups is of ethical importance in an environment where few services are available, and will enhance retention rates in the study. At study assessment visits each child will receive a comprehensive battery of developmental testing, which most families would have to face long wait lists to access. At each assessment the caregiver will be provided with a narrative summary of developmental testing results which they can share with other care providers. Based on our ongoing experience with the Global Brain R21 such clinical information is highly valued by the caregiver and will assist with study retention. Informed consent documents for the study will also include a statement that the study will be posted on ClinicalTrials.gov, with a brief explanation given that this will be unfamiliar to most participants in the study. The participant will be given the opportunity to ask questions.

The child’s parent/legal guardian who signs the consent form must be legally authorized to sign informed consent on behalf of the child. Verbal assent from children will not be sought as per University of Cape Town Policy (study site and primary IRB) the earliest age at which assent is recommended is 7 years, and all children in this study will be 6 years and younger. Verification of comprehension of the consent form will be accomplished by providing participants with a one-page summary sheet of the informed consent information and asking participants to recall central points in the consent process, including the purpose of the study, study procedures involved, and potential risks. This procedure will also provide an opportunity to clarify any points of confusion. Participants will be reminded that they can stop participating at any time or refuse to attend any portion of the study or skip any question in an assessment without penalty.

1. **Privacy and Confidentiality**

**Confidentiality.** Confidentiality is of critical importance, and we will take many precautions to protect against the possibility of a breach of confidentiality. The following precautions will protect the privacy of participants and maintain confidentiality of research data: (1) All study staff will be well trained and will receive ongoing supervision in confidentiality and data security procedures, specifically in ethical conduct, confidentiality protection, review of medical records, mandated reporting, and other topics of human participant protection. In addition, the importance of confidentiality will be emphasized with clinical staff who may have knowledge about patients’ eligibility or participation in the study. (2) As part of the consent procedure, participants will be informed of the limits of confidentiality (i.e., reporting of imminent harm to self or others) and mandated reporting requirements (i.e., reporting of child abuse). (3) Privacy will be maintained by conducting all interviews, discussions, study assessments, and intervention sessions in closed and private rooms. (4) Each participant will be assigned a unique study ID number, and all data will be coded with ID numbers only, not names. The key linking participant names and ID numbers will be stored in a separate password protected document on an encrypted, password protected computer at the University of Cape Town (UCT) study office, to which only the UCT Co-PI and Project Coordinator will have access. Data sent to Duke University will contain only code numbers, and Duke University staff will not have the means to link a participant’s code with their identity. (5) Video study data will be transferred to a secure, HIPAA-compliant project folder on Box.com at Duke University through Box Sync, and deleted from the tablets after confirmation of successful data transfer. (6) Audio files from qualitative interviews will be stored on password-protected, encrypted computers until they are transcribed and translated, and destroyed when process is complete. Transcripts will be transferred to Duke via Box.com. (7) Assessment data will be entered into REDCap (Research Electronic Data Capture), a secure, HIPAA-compliant web-based interface and will be downloaded by Duke staff for storage on a protected server. (8) Paper data collection forms will be securely stored in locked file cabinets in locked offices. Electronic data documents (including interview transcripts) will be saved on password protected, encrypted computers and secure servers. (9) Locator data and consent forms will be stored separately from coded study data. (10) Access to data storage areas and computers will be restricted. (11) All data will be downloaded from Box.com and REDCap and stored on a secure server approved by Duke University for use with sensitive data, accessible only through encrypted, password-protected computers and secure VPN. Data will be deleted off of Box.com, tablets, and local servers after transfer to the protected server. (12) Analysis will occur only on coded data. (13) Data will only be stored for as long as necessary to complete the study, and for adherence to IRB regulations. (14) The information gathered will be used only for scientific, educational, or instructional purposes. Thus, while we acknowledge that a breach of confidentiality is possible, the likelihood is very low. (15) An anonymized dataset will be prepared for public sharing.

Except under circumstances covered under the mandated child abuse reporting laws, and/or situations in which the child and/or a caregiver is judged clinically to be a danger to themselves or others, no information about the child or family will be shared with any individual or agency without prior written consent. Child abuse: Participants will be informed of reporting laws for child abuse and neglect, verbally and in the written consent form. Specifically, the consent form will read: "This protection, however, does not prohibit the investigator from voluntarily reporting information about suspected or known sexual or physical abuse of a child or a subject’s threatened violence to self or others. If the researchers learn that you or someone with whom you are involved is in serious danger of harm, they may inform the appropriate agencies". Therefore, a serious, though rare, risk to families is disruption of the home based upon the severity of the abuse or neglect disclosed.

1. **Participant reimbursement**

We are aware of the ethical considerations in providing participant compensation to people who may be living in poverty because providing cash payments can unintentionally coerce participation in research. Thus, we will restrict compensation to cover the basic costs of participation by providing reimbursement at each study visit (ZAR 150, approx. $8) that is conducted in-person. When participants come into the clinic for in-person evaluation; which are longer time commitments the following amount will be provided: ZAR 300, approx. $20.

**Additional precautions related to in-person assessment and coaching due to COVID-19 (if necessary):**

If the proposal is funded, baseline assessments and coaching sessions are expected to begin in February 2023. We cannot predict the impact of COVID in South Africa at that time. If South Africa is in full lockdown, adjustments in assessments and coaching approach will be required for the duration of the lockdown period. Prior to any protocol adjustments the PI will discuss options/approach with the NIH Program Officer, and protocol amendments will be submitted to the University of Cape Town Human Research Ethics Committee (HREC) and the Duke IRB. If in full lockdown, some data collection (i.e., consent, qualitative interviews, VABS-3, caregiver surveys), and coaching can occur remotely (i.e., via Zoom/phone calls).^1^ However, other activities (i.e., Griffiths III, ADOS-2) that need to occur in-person would be postponed until full lockdown restrictions are lifted. All COVID precautions mandated by the University of Cape Town (study site), the city of Cape Town, and the Education Department will be followed for the duration of the proposed study (i.e., mask mandates, social distancing, hand washing, pre-visit screening, staff vaccinations). Study staff will take the utmost care to ensure all rooms and study materials are thoroughly cleaned in preparation for each study visit, as well as ensuring that all materials are ready for each study visit. If a participant has forgotten their facemask, or their facemask is in poor condition, they will be issued a new mask and will be asked to put it on before entering the building. Participants will be asked during the pre-visit phone call to only bring necessary items to their study visit. Study staff will be required to wear gloves as well as both a mask and protective face shield during all observational assessments and coaching activities that require study staff to be within 6 feet of the participant. In addition, staff will be required to sanitize their hands before and after each observation/coaching session.

1. **Next Steps and Impact**

This proposed project will inform efforts to ensure scalable, targeted, high quality early autism intervention reach the majority of children and families who need it globally. In addition, data on the feasibility and impact of a scalable NDBI coaching approach in an environment with significant contextual challenges will inform tailoring of early autism intervention to diverse communities globally. In the proposed project, we test a feasible, contextually appropriate, non-specialist delivered caregiver coaching NDBI, that can be integrated into existing systems in Africa. We identify determinants that can inform scale-up. Research capacity building will facilitate a next-step regional early autism intervention clinical trial, and inform global efforts to ensure early autism intervention reaches the majority of children who need it.^1, 51, 104, 105^

REFERENCES

1. WHO. Meeting Report: Autism Spectrum Disorders & Other Developmental Disorders: From Raising Awareness to Building Capacity. World Health Organization, 2013.
2. Estes A, Munson J, Rogers SJ, Greenson J, Winter J, Dawson G. Long-Term Outcomes of Early Intervention in 6-Year-Old Children with Autism Spectrum Disorder. Journal of the American Academy of Child Adolescent Psychiatry 2015;54(7):580-7; PMCID: PMC4475272.
3. Cidav Z, Munson J, Estes A, Dawson G, Rogers SJ, Mandell D. Cost Offset Associated with Early Start Denver Model for Children with Autism. Journal of the American Academy of Child Adolescent Psychiatry. 2017;56(9):777-83; PMCID: PMC7007927.
4. Franz L, Chambers N, von Isenburg M, de Vries PJ. Autism spectrum disorder in sub-Saharan Africa: A comprehensive scoping review. Autism Research 2017;10(5):723-49. Epub 2017 Mar 7; PMCID: PMC5512111.
5. Kieling C, Baker-Henningham H, Belfer M, Conti G, Ertem I, Omigbodun O, Rohde LA, Srinath S, Ulkuer N, Rahman A. Child and adolescent mental health worldwide: evidence for action. Lancet. 2011;378(9801):1515-25.
6. Hilton CL, Fitzgerald RT, Jackson KM, Maxim RA, Bosworth CC, Shattuck PT, Geschwind DH, Constantino JN. Brief Report: Under-Representation of African Americans in Autism Genetic Research: A Rationale for Inclusion of Subjects Representing Diverse Family Structures. Journal of Autism and Developmental Disorders. 2010;40(5):633-9; PMCID: PMC3645854.
7. Magana S, Parish S, Rose R, Timberlake M, Swaine J. Racial and ethnic disparities in quality of health care among children with autism and other developmental disabilities. Intellectual and Developmental Disabilities. 2012;50(4):287-99.
8. UNICEF. Generation2030 AFRICA: Child demographics in Africa. 2014.
9. Hamdani SU, Huma Z-e-, Suleman N, Akhtar P, Nazir H, Masood A, Tariq M, Koukab A, Salomone E, Pacione L, Brown F, Shire S, Sikander S, Servili C, Wang D, Minhas FA, Rahman A. Effectiveness of a technology-assisted, family volunteers delivered, brief, multicomponent parents; skills training intervention for children with developmental disorders in rural Pakistan: a cluster randomized controlled trial. International Journal of Mental Health Systems. 2021;15(53). doi: 10.1186/s13033-021-00476-w.
10. Pacia C, Holloway J, Gunning C, Lee H. A Systematic Review of Family-Mediated Social Communication Interventions for Young Children with Autism. Review Journal of Autism and Developmental Disorders. 2021:1-27. doi: 10.1007/s40489-021-00249-8; PMCID: PMC8012416.
11. Schreibman L, Dawson G, Stahmer AC, Landa R, Rogers SJ, McGee GG, Kasari C, Ingersoll B, Kaiser AP, Bruinsma Y, McNerney E, Wetherby A, Halladay A. Naturalistic Developmental Behavioral Interventions: Empirically Validated Treatments for Autism Spectrum Disorder. Journal of Autism and Developmental Disorders. 2015;45(8):2411-28; PMCID: PMC4513196.
12. Dawson G, Rogers S, Munson J, Smith M, Winter J, Greenson J, Donaldson A, Varley J. Randomized, controlled trial of an intervention for toddlers with autism: the Early Start Denver Model. Pediatrics. 2010;125(1):e17-23; PMCID: PMC4951085.
13. Guler J, de Vries PJ, Seris N, Shabalala N, Franz L. The importance of context in early autism intervention: A qualitative South African study. Autism 2018;22(8):1005-17. Epub 2017 Sep 15; PMCID: PMC5832543.
14. Franz L, Adewumi K, Chambers N, Viljoen M, Baumgartner JN, de Vries PJ. Providing early detection and early intervention for autism spectrum disorder in South Africa: stakeholder perspectives from the Western Cape province. Journal of Child and Adolescent Mental Health. 2018;30(3):149-65. Epub 2018 Nov 7; PMCID: PMC6301128.
15. Ramseur K, de Vries PJ, Guler J, Shabalala N, Seris N, Franz L. Caregiver descriptions of joint activity routines with young children with autism spectrum disorder in South Africa. Pediatric Medicine. 2019;2. Epub 2019 Mar 13; PMCID: PMC6481954.
16. Makombe CBT, Shabalala N, Viljoen M, Seris N, de Vries PJ, Franz L. Sustainable implementation of early intervention for autism spectrum disorder through caregiver coaching: South African perspectives on barriers and facilitators. Pediatric Medicine. 2019;2; PMCID: PMC6746419.
17. Ndlovu M, Dawood Z, Viljoen M, Seris N, Shabalala N, Harty M, Simmons R, Turner L, de Vries PJ, Franz L. Using the Joint Engagement Rating Inventory (JERI) as a Measurement of Caregiver-Child Interactions in Young Children with Autism Spectrum Disorder in a Low Resource South African Setting. FIC Global Brain Network Meeting2021.
18. Viljoen M, Seris N, Shabalala N, Ndlovu M, de Vries PJ, Franz L. Using Telehealth to coach caregivers of young children with autism spectrum disorder in South Africa [Poster presentation]. FIC Global Brain Network Meeting 2021.
19. Joint Press Statement: Early Childhood Development (Ecd) Function Shift. South African Government Department of Basic Education 2021.
20. Kumm AJ, Viljoen M, de Vries PJ. The Digital Divide in Technologies for Autism: Feasibility Considerations for Low- and Middle-Income Countries. Journal of Autism and Developmental Disorders. 2021:1-14. doi: 10.1007/s10803-021-05084-8; PMCID: PMC8200284.
21. Moullin JC, Dickson KS, Stadnick NA, Rabin B, Aarons GA. Systematic review of the Exploration, Preparation, Implementation, Sustainment (EPIS) framework. Implementation Science 2019;14(1); PMCID: PMC6321673.
22. Mayosi BM, Benatar SR. Health and health care in South Africa--20 years after Mandela. New England Journal of Medicine. 2014;371(14):1344-53.
23. Pickles A, Couteur AL, Leadbitter K, Salomone E, Cole-Fletcher R, Tobin H, Gammer I, Jessica Lowry, Vamvakas G, Byford S, Aldred C, Slonims V, McConachie H, Howlin P, Parr JR, Charman T, Green J. Parent mediated social communication therapy for young children with autism (PACT): long-term follow-up of a randomised controlled trial. Lancet. 2016;388:2501-9. Epub 2016 Oct 25; PMCID: PMC5121131.
24. Wetherby AM, Guthrie W, Woods J, Schatschneider C, Holland RD, Morgan L, Lord C. Parent implemented social intervention for toddlers with autism: an RCT. Pediatrics. 2014;134(6):1084-93. Epub 2014. Nov 3; PMCID: PMC4243066.
25. Sinai-Gavrilov Y, Gev T, Mor-Snir I, Vivanti G, Golan O. Integrating the Early Start Denver Model into Israeli community autism spectrum disorder preschools: Effectiveness and treatment response predictors. Autism. 2020;24(8):2081-93. Epub 2020 Jul 14; PMCID: PMC7543011.
26. Touzet S, Occelli P, Schröder C, Manificat S, Gicquel L, Stanciu R, Schaer M, Oreve M-J, Speranza M, Denis A, Zelmar A, Falissard B, Georgieff N, Bahrami S, Geoffray M-M, Group IS. Impact of the Early Start Denver Model on the cognitive level of children with autism spectrum disorder: study protocol for a randomised controlled trial using a two-stage Zelen design. BMJ open. 2017;7(3):e014730; PMCID: PMC5372147.
27. Lin T-L, Chiang C-H, Ho SY, Wu H-C, Wong C-C. Preliminary clinical outcomes of a short-term low intensity Early Start Denver Model implemented in the Taiwanese public health system. Autism. 2020;24(5):1300-6. Epub 2020 Jan 8.
28. Colombi C, Narzisi A, Ruta L, Cigala V, Gagliano A, Pioggia G, Siracusano R, Rogers SJ, Muratori F, Team PP. Implementation of the Early Start Denver Model in an Italian community. Autism. 2018;22(2):126-33. Epub 2016 Oct 20.
29. Devescovi R, Colonna V, Dissegna A, Bresciani G, Carrozzi M, Colombi C. Feasibility and Outcomes of the Early Start Denver Model Delivered within the Public Health System of the Friuli Venezia Giulia Italian Region. Brain Sciences. 2021;11(9); PMCID: PMC8464931.
30. Li H-H, Li C-L, Gao D, Pan X-Y, DU L, Jia F-Y. [Preliminary application of Early Start Denver Model in children with autism spectrum disorder]. Zhongguo Dang Dai Er Ke Za Zhi. 2018;20(10):793-8; PMCID: PMC7389043.
31. Zhou B, Xu Q, Li H, Zhang Y, Wang Y, Rogers SJ, Xu X. Effects of Parent-Implemented Early Start Denver Model Intervention on Chinese Toddlers with Autism Spectrum Disorder: A Non-Randomized Controlled Trial. Autism Research. 2018;11(4):654-66. Epub 2018 Feb 7.
32. Tateno Y, Kumagai K, Monden R, Nanba K, Yano A, Shiraishi E, Teo AR, Tateno M. The Efficacy of Early Start Denver Model Intervention in Young Children with Autism Spectrum Disorder Within Japan: A Preliminary Study. Soa Chongsonyon Chongsin Uihak. 2021;32(1):35-40; PMCID: PMC7788666.
33. Holzinger D, Laister D, Vivanti G, Barbaresi WJ, Fellinger J. Feasibility and Outcomes of the Early Start Denver Model Implemented with Low Intensity in a Community Setting in Austria. Journal of Developmental and Behavioral Pediatrics. 2019;40(5):354-63.
34. Shi B, Wu W, Dai M, Zeng J, Luo J, Cai L, Wan B, Jing J. Cognitive, Language, and Behavioral Outcomes in Children With Autism Spectrum Disorders Exposed to Early Comprehensive Treatment Models: A Meta-Analysis and Meta-Regression. Frontiers in Psychiatry 2021;12; PMCID: PMC8350444.
35. Fuller EA, Oliver K, Vejnoska SF, Rogers SJ. The Effects of the Early Start Denver Model for Children with Autism Spectrum Disorder: A Meta-Analysis. Brain Sciences. 2020;10(6); PMCID: PMC7349854.
36. Roche L, Adams D, Clark M. Research priorities of the autism community: A systematic review of key stakeholder perspectives. Autism. 2021;25(2):336-48.
37. Schuck RK, Tagavi DM, Baiden KMP, Dwyer P, Williams ZJ, Osuna A, Ferguson EF, Muñoz MJ, Poyser SK, Johnson JF, Vernon TW. Neurodiversity and Autism Intervention: Reconciling Perspectives Through a Naturalistic Developmental Behavioral Intervention Framework. Journal of Autism and Developmental Disorders. 2021. Epub Online ahead of print.
38. Reichow B, Servili C, Yasamy MT, Barbui C, Saxena S. Non-specialist psychosocial interventions for children and adolescents with intellectual disability or lower-functioning autism spectrum disorders: a systematic review. PLoS Med. 2013;10(12). Epub 2013 Dec 17; PMCID: PMC3866092.
39. Wong VCN, Kwan QK. Randomized controlled trial for early intervention for autism: a pilot study of the Autism 1-2-3 Project. Journal of Autism and Developmental Disorders.40(6):677-88.
40. Tsang SKM, Shek DTL, Lam LL, Tang FLY, Cheung PMP. Brief report: application of the TEACCH program on Chinese pre-school children with autism--Does culture make a difference? Journal of Autism and Developmental Disorders. 2007;37(2):390-6.
41. Divan G, Vajaratkar V, Cardozo P, Huzurbazar S, Verma M, Howarth E, Emsley R, Taylor C, Patel V, Green J. The Feasibility and Effectiveness of PASS Plus, A Lay Health Worker Delivered Comprehensive Intervention for Autism Spectrum Disorders: Pilot RCT in a Rural Low- and Middle-Income Country Setting. Autism Research. 2019;12(2):328-39. Epub 2018 Aug 10.
42. Schleiff MJ, Aitken I, Alam MA, Damtew ZA, Perry HB. Community health workers at the dawn of a new era: 6. Recruitment, training, and continuing education. Health Research Policy and Systems. 2021;12(19); PMCID: PMC8506097.
43. Black MM, Walker SP, Fernald LCH, Andersen CT, DiGirolamo AM, Lu C, McCoy DC, Fink G, Shawar YR, Shiffman J, Devercelli AE, Wodon QT, Vargas-Barón E, Grantham-McGregor S, Lancet Early Childhood Development Series Steering Committee. Early childhood development coming of age: science through the life course. Lancet. 2017;389(10064):77-90. Epub 2016 Oct 4; PMCID: PMC5884058.
44. Richter LM, Daelmans B, Lombardi J, Heymann J, Boo FL, Behrman JR, Lu C, Lucas JE, Perez-Escamilla R, Dua T, Bhutta ZA, Stenberg K, Gertler P, Darmstadt GL, Paper 3 Working Group and the Lancet Early Childhood Development Series Steering Committee. Investing in the foundation of sustainable development: pathways to scale up for early childhood development. Lancet. 2017;389(10064):103-18. Epub 2016 Oct 4; PMCID: PMC5880532.
45. Jaskiewicz W, Tulenko K. Increasing community health worker productivity and effectiveness: a review of the influence of the work environment. Human Resources for Health 2012;10; PMCID: PMC3472248.
46. Smith S, Deveridge A, Berman J, Negin J, Mwambene N, Chingaipe E, Ritchie LMP, Martiniuk A. Task shifting and prioritization: a situational analysis examining the role and experiences of community health workers in Malawi. Human Resources for Health. 2014;12; PMCID: PMC4014628.
47. Okyere E, Mwanri L, Ward P. Is task-shifting a solution to the health workers' shortage in Northern Ghana? PLoS One. 2017;12(3). Epub 2017; PMCID: PMC5373592.
48. Springer PE, van Toorn R, Laughton B. Characteristics of children with pervasive developmental disorders attending a developmental clinic in the Western Cape Province, South Africa. South African Journal of Child Health. 2013;7(3):95-9.
49. Pillay S, Duncan M, Vries PJd. Autism in the Western Cape province of South Africa: Rates, socio-demographics, disability and educational characteristics in one million school children. Autism. 2021;25(4):1076-89. Epub 2020 Dec 17.
50. Aarons GA, Hurlburt M, Horwitz SM. Advancing a conceptual model of evidence-based practice implementation in public service sectors. Adm Policy Ment Health. 2011;38(1):4-23; PMCID: PMC3025110.
51. Republic of South Africa. National Integrated Early Childhood Development Policy. In: Development DoS, editor. Republic of South Africa Pretoria: Government Printers; 2015.
52. Rieder A, Viljoen M, Seris N, Shabalala N, Ndlovu M, Turner L, Simmons R, de Vries PJ, Franz L. Pilot Study to Improve Access to Early Intervention for Autism in Africa. Under Review.
53. Campbell K, Carpenter KL, Hashemi J, Espinosa S, Marsan S, Borg JS, Chang Z, Qiu Q, Vermeer S, Adler E, Tepper M, Egger HL, Baker JP, Sapiro G, Dawson G. Computer vision analysis captures atypical attention in toddlers with autism. Autism. 2019;23(3):619-28. Epub 2018 Mar 29; PMCID: PMC6119515.
54. Campbell K, Carpenter KLH, Espinosa S, Hashemi J, Qiu Q, Tepper M, Calderbank R, Sapiro G, Egger HL, Baker JP, Dawson G. Use of a Digital Modified Checklist for Autism in Toddlers - Revised with Follow-up to Improve Quality of Screening for Autism. Journal of Pediatrics. 2017 183:133-9. Epub 2017 Feb 1; PMCID: PMC5397992.
55. Carpenter KLH, Hahemi J, Campbell K, Lippmann SJ, Baker JP, Egger HL, Espinosa S, Vermeer S, Sapiro G, Dawson G. Digital Behavioral Phenotyping Detects Atypical Pattern of Facial Expression in Toddlers with Autism. Autism Research. 2021;14(3):488-99. Epub 2020 Sep 14; PMCID: PMC7920907.
56. Egger HL, Dawson G, Hashemi J, Carpenter KLH, Espinosa S, Campbell K, Brotkin S, Schaich-Borg J, Qiu Q, Tepper M, Baker JP, Jr RAB, Sapiro G. Automatic emotion and attention analysis of young children at home: a ResearchKit autism feasibility study. NPJ Digital Medicine. 2018;1. Epub eCollection 2018; PMCID: PMC6550157.
57. Hashemi J, Tepper M, Spina TV, Esler A, Morellas V, Papanikolopoulos N, Egger H, Dawson G, Sapiro G. Computer vision tools for low-cost and noninvasive measurement of autism-related behaviors in infants. Autism Research and Treatment 2014;2014. Epub 2014 Jun 22; PMCID: PMC4090521.
58. Chang Z, Martino JMD, Aiello R, Baker J, Carpenter K, Compton S, Davis N, Eichner B, Espinosa S, Flowers J, Franz L, Harris A, Howard J, Perochon S, Perrin EM, Babu PRK, Spanos M, Sullivan C, Walter BK, Kollins SH, Dawson G, Sapiro G. Computational Methods to Measure Patterns of Gaze in Toddlers with Autism Spectrum Disorder. JAMA Pediatrics. 2021;175(8):827-36; PMCID: PMC8077044 (available on 2022-04-26).
59. Perochon S, Martino MD, Aiello R, Baker J, Carpenter K, Chang Z, Compton S, Davis N, Eichner B, Espinosa S, Flowers J, Franz L, Gagliano M, Harris A, Howard J, Kollins SH, Perrin EM, Raj P, Spanos M, Walter B, Sapiro G, Dawson G. A scalable computational approach to assessing response to name in toddlers with autism. Journal of Child Psychology and Psychiatry. 2021;62(9):1120-31. Epub 2021 Feb 28; PMCID: PMC8397798 (available on 2022-09-01).
60. The Times Higher Education. World University Rankings 2021 [cited 2020 October 5]. Available from: Times Higher Education website.
61. Duke Global Health Institute. Where We Work [cited 2020 October 5]. Available from: Duke Global Health website.
62. Lachman A, Berg A, Ross F, Pentecost M. Infant mental health in southern Africa: nurturing a field. The Lancet. 2021;398(10303):P835-P6.
63. Rogers SJ, Vismara L, Dawson G, Stahmer A. Help Is in Your Hands: A Web-Based Tool to Help Parents Build Their Toddlers’ Social and Communication Development. 2017.
64. Nielsen M, Haun D, Kärtner J, Legare CH. The persistent sampling bias in developmental psychology: A call to action. Journal of Experimental Child Psychology. 2017;162:31-8. Epub 2017 May 30.
65. Ndlovu, M. Towards Naturalistic Developmental Behavioural Interventions for Autism in Africa: Content and Contexts of Caregiver-Child Dyadic Interactions in Low-Resource South African Environments Expected date of submission: Feb 2022.
66. Dawood Z. Describing the outcomes of a caregiver mediated intervention on children with Autism Spectrum Disorder in Cape Town, South Africa. Masters in Speech and Language Pathology. University of Cape Town. Expected date of submission: June 2022.
67. Adamson LB. The joint engagement rating inventory (Technical Report 25, 2nd ed.). Atlanta, GA: Georgia State University 2018.
68. Adamson LB, Bakeman R, Suma K, Robins DL. An Expanded View of Joint Attention: Skill, Engagement, and Language in Typical Development and Autism. Child Development 2019;90(1):e1-e18. Epub 2017 Oct 9; PMCID: PMC5891390.
69. Rogers SJ, Dawson G. Early Start Denver Model for Young Children with Autism Promoting Language, Learning, and Engagement. New York: Guilford Press; 2010.
70. Sparrow SS, Cichetti DV, Saulnier CS. Vineland Adaptive Behavior Scales, Third Edition. San Antonio, TX: Pearson; 2016.
71. Green E, Stroud L, Bloomfield S, Cronje J, Foxcroft C, Hurter K, Lane H, Marais R, Marx C, McAlinden P, O’Connell R, Paradice R, Venter D. Griffiths Scales of Child Development, 3^rd^ Edition. Western Psychological Services; 2016.
72. Viljoen, M. Using Telehealth to coach caregivers of young children with autism spectrum disorder in South Africa: intervention adaptation, feasibility and early signals of change. PhD in Neuroscience (Psychiatry). University of Cape Town. Expected date of submission: May 2022.
73. Dingfelder HE, Mandell DS. Bridging the research-to-practice gap in autism intervention: an application of diffusion of innovation theory. Journal of Autism and Developmental Disorders. 2011;41(5):597-609; PMCID: PMC3077435.
74. Gomez E, Drahota A, Stahmer AC. Choosing strategies that work from the start: A mixed methods study to understand effective development of community-academic partnerships. Action Research (Lond). 2021;19(2):277-300. Epub 2018 May 19; PMCID: PMC8447887 (available on 2021-12-01).
75. Kuznetsova OM, Tymofyeyev Y. Preserving the allocation ratio at every allocation with biased coin randomization and minimization in studies with unequal allocation. Statistics in Medicine. 2012;31(8):701-23. Epub 2011 Dec 12.
76. Constantino JN, Gruber CP. Social responsiveness scale (SRS) manual. Los Angeles Western Psychological Services; 2005.
77. Schopler E, Reichler RJ, DeVellis RF, Daly K. Toward objective classification of childhood autism: Childhood Autism Rating Scale (CARS). Journal of Autism and Developmental Disorders. 1980;10(1):91-103. doi: 10.1007/BF02408436.
78. Chambers NJ, Wetherby AM, Stronach ST, Njongwe N, Kauchali S, Grinker RR. Early detection of autism spectrum disorder in young isiZulu-speaking children in South Africa. Autism. 2017;21(5):518-26. Epub 2016 Jun 22. doi: 10.1177/1362361316651196.
79. Smith L, Malcolm-Smith S, Vries PJd. Translation and cultural appropriateness of the Autism Diagnostic Observation Schedule-2 in Afrikaans. Autism. 2017;21(5):552-63. Epub 2016 May 25. doi: 10.1177/1362361316648469.
80. Chatham CH, Taylor KI, Charman T, D'ardhuy XL, Eule E, Fedele A, Hardan AY, Loth E, Murtagh L, Rubido MdV, Caceres ASJ, Sevigny J, Sikich L, Snyder L, Tillmann JE, Ventola PE, Walton-Bowen KL, Wang PP, Willgoss T, Bolognani F. Adaptive behavior in autism: Minimal clinically important differences on the Vineland-II. Autism Research. 2017;11(2):270-83. Epub 2017 Sep 21; PMCID: PMC5997920.
81. Frost KM, Brian J, Gengoux GW, Hardan A, Rieth SR, Stahmer A, Ingersoll B. Identifying and measuring the common elements of naturalistic developmental behavioral interventions for autism spectrum disorder: Development of the NDBI-Fi. Autism. 2020;24(8):2285-97. Epub 2020 Jul 30; PMCID: PMC7541530.
82. Abidin RR. Manual for the parenting stress index (3rd ed.). Odessa, FL: Psychological Assessment Resources; 1995.
83. Johnston C, Mash EJ. A Measure of Parenting Satisfaction and Efficacy. Journal of Clinical Child Psychology. 1989;18(2):167-75. Epub 2010 Jun 7.
84. Baron RM, Kenny DA. The moderator-mediator variable distinction in social psychological research: Conceptual, strategic, and statistical considerations. Journal of Personality and Social Psychology. 1986;51(6):1173-82.
85. Glaser BG, Anselm L S. The Discovery of Grounded Theory: Strategies for Qualitative Research Chicago: Aldine 1967.
86. Ramsey SD, Willke RJ, Glick H, Reed SD, Augustovski F, Jonsson B, Briggs A, Sullivan SD. Cost-effectiveness analysis alongside clinical trials II-An ISPOR Good Research Practices Task Force report. Value Health. 2015;18(2):161-72. doi: 10.1016/j.jval.2015.02.001.
87. Sanders GD, Neumann PJ, Basu A, Brock DW, Feeny D, Krahn M, Kuntz KM, Meltzer DO, Owens DK, Prosser LA, Salomon JA, Sculpher MJ, Trikalinos TA, Russell LB, Siegel JE, Ganiats TG. Recommendations for Conduct, Methodological Practices, and Reporting of Cost-effectiveness Analyses: Second Panel on Cost-Effectiveness in Health and Medicine. JAMA 2016;316(10):1093-103. doi: 10.1001/jama.2016.12195.
88. Shepard DS. Cost-effectiveness in Health and Medicine. By M.R. Gold, J.E Siegel, L.B. Russell, and M.C. Weinstein (eds). New York: Oxford University Press, 1996. The Journal of Mental Health Policy and Economics 1999;2(2):91-2.
89. Powell BJ, Waltz TJ, Chinman MJ, Damschroder LJ, Smith JL, Matthieu MM, Proctor EK, Kirchner JE. A refined compilation of implementation strategies: results from the Expert Recommendations for Implementing Change (ERIC) project. Implementation Science. 2015;10(21); PMCID: PMC4328074.
90. Brown AF, Ma GX, Miranda J, Eng E, Castille D, Brockie T, Jones P, Airhihenbuwa CO, Farhat T, Zhu L, Trinh-Shevrin C. Structural Interventions to Reduce and Eliminate Health Disparities. American Journal of Public Health. 2019;109(S1):S78-S; PMCID: PMC6356131.
91. Brannan AM, Heflinger CA, Bickman L. The Caregiver Strain Questionnaire: Measuring the Impact on the Family of Living with a Child with Serious Emotional Disturbance Journal of Emotional and Behavioral Disorders. 1997;5(4):212-22.
92. Woods-Jaeger BA, Kava CM, Akiba CF, Lucid L, Dorsey S. The art and skill of delivering culturally responsive trauma-focused cognitive behavioral therapy in Tanzania and Kenya. Psychological Trauma. 2017;9(2):230-8. Epub 2016 Jul 14; PMCID: PMC5237406.
93. Drahota A, Meza RD, Brikho B, Naaf M, Estabillo JA, Gomez ED, Vejnoska SF, Dufek S, Stahmer AC, Aarons GA. Community-Academic Partnerships: A Systematic Review of the State of the Literature and Recommendations for Future Research. Milbank Quarterly. 2016;94(1):163-214; PMCID: PMC4941973.
94. Aarons GA, Ehrhart MG, Farahnak LR. The Implementation Leadership Scale (ILS): development of a brief measure of unit level implementation leadership. Implementation Science. 2014;9(1); PMCID: PMC4022333.
95. Ehrhart MG, Aarons GA, Farahnak LR. Assessing the organizational context for EBP implementation: the development and validity testing of the Implementation Climate Scale (ICS). Implementation Science. 2014;9; PMCID: PMC4210525.
96. Nadeem E, Jaycox LH, Kataoka SH, Langley AK, Stein BD. Going to Scale: Experiences Implementing a School-Based Trauma Intervention. School Psychology Review. 2011;40(4):549-68; PMCID: PMC4917015.
97. Rogers SJ, Vismara LA, Dawson G. Coaching Parents of Young Children with Autism. Promoting Connection, Communication, and Learning. New York: Guilford Press; 2021.
98. Nadeem E, Ringle V. De-adoption of an evidence-based trauma intervention in schools: A retrospective report from an urban school district. School Mental Health. 2016;8(1):132-43. Epub 2016 Jan 23; PMCID: PMC5538780.
99. Murray LK, Skavenski S, Michalopoulos LM, Bolton PA, Bass JK, Familiar I, Imasiku M, Cohen J. Counselor and client perspectives of Trauma-focused Cognitive Behavioral Therapy for children in Zambia: a qualitative study. Journal of Child and Adolescent Psychology. 2014;43(6):902-14. Epub 2014 Jan 8; PMCID: PMC4087094.
100. Creswell JW, Plano Clark VL. Designing and conducting mixed methods research (2nd ed.). Los Angeles: SAGE Publications; 2011.
101. Schlebusch L, Chambers NJ, Dawson-Squibb JJ, Harty M, Franz L, de Vries PJ. Challenges and opportunities of implementing early interventions for autism spectrum disorders in resource-limited settings: A South African example. In: Hodes H, Shur-Fen Gau S, de Vries PJ, editors. Starting at the beginning: Laying the foundation for lifelong mental health London: Elsevier Academic Press; 2020. p. 99-132.
102. Chambers N, Dawson-Squibb J-J, Franz L, Harty M, Schlebusch L, de Vries PJ, editors. African Autism Treatment Network (AATN): Lessons Learned. SA-ACAPAP Sustaining Development: Investing in child and adolescent mental health; 2019; Johannesburg, South Africa.
103. Makombe CB, Shabalala N, Viljoen M, Seris N, de Vries PJ, Franz L, editors. Barriers and facilitators to implementing a caregiver coaching early autism spectrum disorder intervention in South Africa. INSAR; 2019; Montreal, Canada.
104. Salomone E, Pacione L, Shire S, Brown FL, Reichow B, Servili C. Development of the WHO Caregiver Skills Training Program for Developmental Disorders or Delays. Frontiers in Psychiatry. 2019;10. Epub eCollection 2019; PMCID: PMC6859468.
105. Salomone E, Settanni M, McConachie H, Suma K, Ferrara F, Foletti G, Salandin A, Team WC, Servili C, Adamson LB. Pilot Randomized Controlled Trial of the WHO Caregiver Skills Training in Public Health Services in Italy 2021; Journal of Autism and Developmental Disorders. Epub ahead of print.
